# Case Report: A Novel *KMT2E* Splice Site Variant as a Cause of O'Donnell-Luria-Rodan Syndrome in a Male Patient

**DOI:** 10.3389/fped.2022.822096

**Published:** 2022-02-22

**Authors:** Zixuan Cao, Chunli Wang, Jing Chen, Hu Guo, Chunfeng Wu, Gang Zhang, Le Ding

**Affiliations:** ^1^Department of Neurology, Children's Hospital of Nanjing Medical University, Nanjing, China; ^2^Nanjing Key Laboratory of Pediatrics, Children's Hospital of Nanjing Medical University, Nanjing, China

**Keywords:** O'Donnell-Luria-Rodan's syndrome, *KMT2E*, epilepsy, splice variant, abnormal mRNA splicing

## Abstract

**Background:**

O'Donnell-Luria-Rodan (ODLURO) syndrome is an autosomal dominant systemic disorder characterized by global developmental delay caused by mutations in the *KMT2E* gene. The aim of this study was to investigate the role of *KMT2E* mutations as a cause of ODLURO syndrome in a Chinese boy.

**Methods:**

We reported the clinical course of a Chinese boy who was diagnosed with ODLURO syndrome by the whole exome sequencing. We extracted genomic DNA of the proband and parents, gene variations were screened using whole-exome sequencing, followed by validation using direct Sanger sequencing. The effect of mRNA splicing variants were analyzed through a minigene splice assay and *in vitro* reverse transcription PCR (RT-PCR).

**Results:**

The proband presented with recurrent seizures and developmental delay. Using genetic analysis, we identified that the proband carried a *de novo* heterozygous splicing variant (c.1248+1G>T) in the *KMT2E* gene. *In vivo* transcript analysis showed that the proband did not carry any *KMT2E* mRNA transcript, while a specific exon11-exon13 (440 bp) transcript was detected in the unaffected parents. The *in vitro* minigene splice assay conducted in HEK293 cells confirmed that the c.1248+1G>T variant resulted in exon 12 skipping, which in turn caused an alteration in *KMT2E* mRNA splicing. The mutant transcript created a premature stop codon at the 378 amino acid position that could have been caused nonsense-mediated mRNA decay (NMD).

**Conclusion:**

We verified the pathogenic effect of the *KMT2E* c.1248+1G>T splicing variant, which disturbed normal mRNA splicing and caused mRNA decay. Our findings suggest that splice variants play an important role in the molecular basis of ODLURO, and that careful molecular profiling of these patients could play an essential role in tailoring of personalized treatment options soon.

## Introduction

O'Donnell-Luria-Rodan (ODLURO) syndrome (OMIM: 618512) is an autosomal dominant neurodevelopmental disorder characterized by global development delay (1). The symptoms of ODLURO syndrome include variably developmental and speech delay, autism, seizures, hypotonia, and dysmorphic features. ODLURO syndrome is caused by a heterozygous mutation in the *KMT2E (MLL5)* gene (OMIM: 608444) on chromosome 7q22. *KMT2E* encodes a member of the lysine N-methyltransferase 2 (KMT2) family (2). This family of enzymes plays a vital role in regulating post-translational methylation of histone 3 on lysine 4 (H3K4) (3). H3K4 methylation is required to maintain open chromatin states for normal regulation of transcription. There are at least eight known monogenic neurodevelopment disorders where regulation of H3K4 methylation is impaired (4). In addition to these Mendelian disorders, dysregulated H3K4 methylation is believed to play a role in the pathogenesis of schizophrenia and autism (5). KMT2E participates in a variety of biological processes, including cell cycle progression, maintenance of genomic stability, and sperm formation (6, 7). *KMT2E* dysfunction is also associated with several non-communicable diseases, including cancer, neurological disorders, and coronary artery disease.

In this study, we describe the case of a male patient with recurrent seizures and developmental delay diagnosed with ODLURO syndrome caused by a novel *KMT2E* splice variant (c.1248+1G>T) associated with abnormal mRNA splicing and nonsense-mediated mRNA decay (NMD). This case study suggests that *KMT2E* splice variants play an important role in the molecular basis of ODLURO syndrome.

## Materials and Methods

### Clinical Presentation

**Figure 1:**
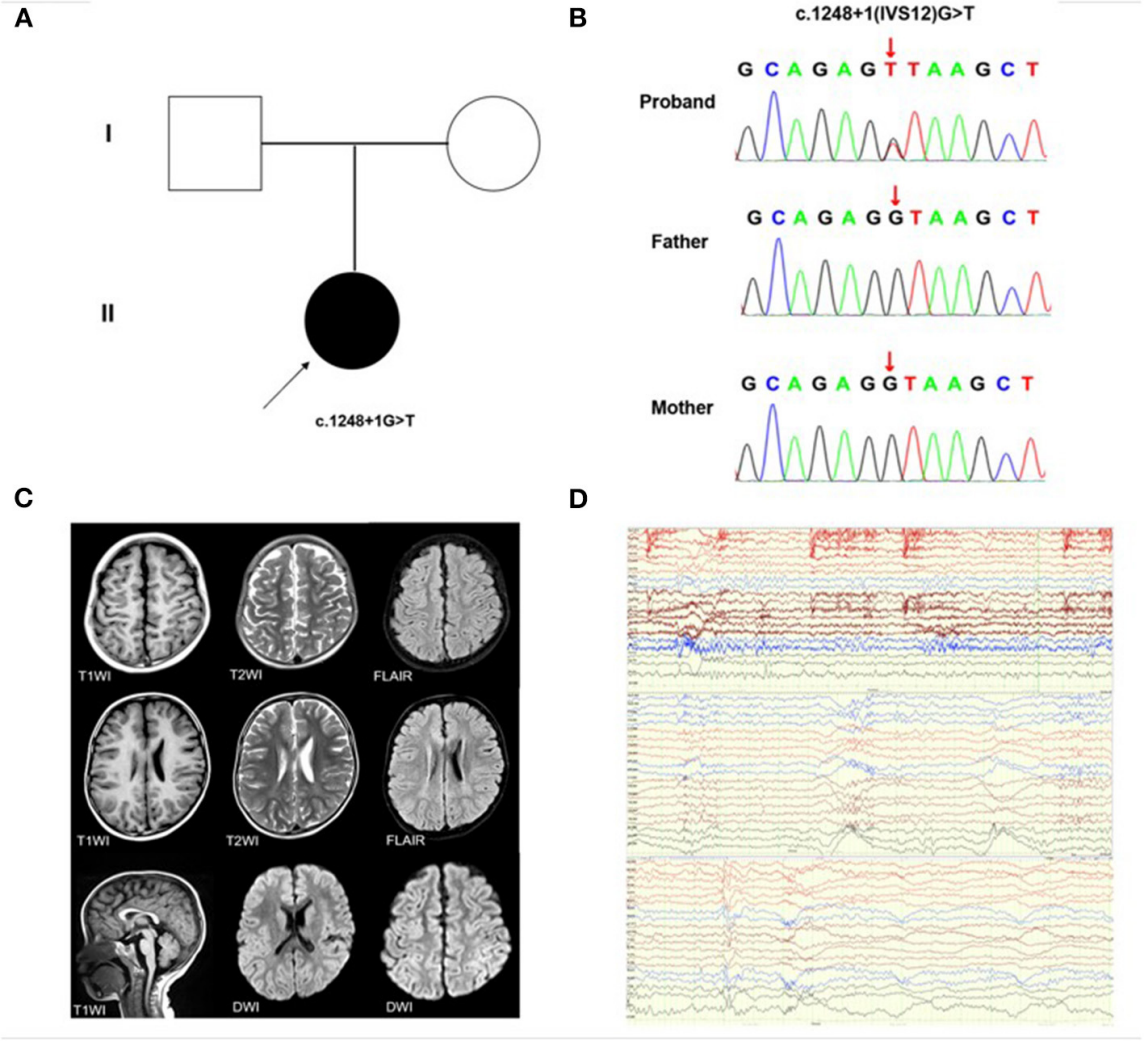
*KMT2E* gene variant and auxiliary examination of the patient. **(A)** Schematic presentation of the familial pedigrees. Males and females are designated by squares and circles, respectively. The shapes filled with shadow indicate affected individuals with the *KMT2E* variant; the arrow indicates the proband. **(B)** Direct sequencing identified the novel *KMT2E* (c.1248+1G>T) variant **(C)** Brain MRI of the patient showing delayed myelination **(D)** The EEG reports of the patients, from top to bottom, representing the background slow wave, general fast wave rhythm during sleep, and general fast wave rhythm during the waking state.

The proband was a 3 year, 9 month old boy, admitted to hospital with a 1-week history of repeated focal tonic-clonic seizures (FCS), including loss of contact, stiff limbs, and cyanosis, initial fever was absent. The onset of seizures was usually during sleep. The proband had a head circumference of approximately 43 cm, height 100 cm, weight 16.5 kg, and no facial dysmorphisms (such as a large forehead, deep-set eyes, and full cheeks) were present. His skin is free of café-au-lait spots or hyperpigmentation. The proband presented with hypotonia of the right upper arm, physiological reflexes were present, but pathological reflexes were not elicited. The patient was born at full-term via normal vaginal delivery without birth related distress or dysmorphia. Perinatal history and pregnancy were both unremarkable. The proband did not undergo any screening for malformation prior to the onset of the disease. Familial antecedents were negative. No obvious abnormality was found in routine blood examination, biochemistry, hematuria metabolism screening, auto-immune encephalitis anti-body detection, cerebrospinal fluid examination, electrocardiogram, chest X-ray, cardiac color ultrasound, urinary system, and abdominal B-ultrasound examination. Magnetic Resonance Imaging (MRI) revealed that the head and spine were normal. Video Electroencephalogram (EEG) showed a cluster of seizures during the awake period and an isolated seizure during sleep. Spasms occurred when the head was tilted back and nodding, and a wide-ranging fast-wave rhythm was present during the seizure. Treatment was initiated with valproic acid (VPA) 36 mg/kg/d and topiramate (TPM) 3 mg/kg/d. The patient convulsed once more at the age of five, and we increased the TPM dose to 3.5 mg/kg/d. The most recent brain MRI showed delayed myelination (Figure 1C). Re-examination of the EEG showed that the background activity slowed down during sleep, and there were multiple sharp spikes and slow waves in the center and midline area bilaterally (Figure 1D). At the most recent outpatient follow-up, the proband hadn't had seizures in a year, and the EEG still showed background slow waves. His parents described his preference for being alone, often refusing to communicate and demonstrating social difficulties, distinct speech delay, and intellectual disability (ID) were present. No scales had been used to accurately assess intelligence or mental status before or after his onset.

### Whole-Exome Sequencing

Genomic DNA was extracted from peripheral blood using standard procedures. The study was performed according to the ethical guidelines of the Children's Hospital of Nanjing Medical University. Written informed consent was obtained from all participants. WES was performed on an Illumina HiSeq 2000 (Bio-Rad, Hercules, CA, USA) using 2× 100-bp paired-end reads. Variants with allele frequencies higher than 1%were filtered out. The minor allele frequency (MAF) was annotated using databases dbSNP, 1000 Genomes MAF (Chinese), ExAC, Genome Aggregation Database (gnomAD), and an in-house MAF database. The candidate variants were validated using Sanger sequencing, and the pathogenicity of variants was annotated according to the American College of Medical Genetics and Genomics (ACMG) standards and guidelines.

### Direct Sequencing of the *KMT2E* Gene

To amplify exon 12 of the *KMT2E* gene, primer pairs were designed (F:AAGTTATGGTACTGATGTAACCAGG; R:GTAGTAGCACTGCCCTCTCTTTAT). The PCR mixtures contained 1.5 μl primers, 2.0 μl DNA, 12.5 μl 2 × Taq Master Mix (Vazyme Biotech Co., Ltd., Nanjing, China), and 9 μl ddH_2_O in a total volume of 25 μl. Cycling conditions included a pre-denaturation step at 94°C for 5 min, followed by 34 cycles at 94°C for 30 s, 59°C for 30 s, and 72°C for 30 s, and a final extension at 72°C for 5 min. The PCR products were purified and then sequenced using the BigDye Terminator v3.1 Cycle Sequencing Kit (Applied Biosystems, Foster City, CA, USA). In addition, 200 healthy, unrelated controls from the Chinese population were screened using Sanger sequencing to exclude novel variants as non-disease associated variations. A *KMT2E* gene variant (GenBank accession number NM_182931) was used as a reference sequence.

### Transcript Analysis *in Vivo*

Total RNA was extracted from peripheral leukocytes of the proband and parents. We used mRNA extracted from a control leukocyte obtained from the blood sample of a healthy volunteer. RNA was isolated using the QIAGEN miRNeasy Mini Kit, and then reverse-transcribed into cDNA by using random hexamers and the SuperScript III transcriptase (Invitrogen). The resulting cDNA was used as a template to amplify a 440-bp product containing exon 11–13 using forward primer *KMT2E*-E12-F (5′-ACCAACAATTTGCTCTTCAAACCTCC-3′) and reverse primer *KMT2E*-E12-R (5′-GCACTCTGGGTTTTCTTTGAGGCA-3′). PCR products were separated on 1.5% agarose and sequenced with an ABI3130genetic analyzer (Applied Biosystems).

### Minigene Plasmid Construction and Site-Directed Mutagenesis

We used the pSPL3 minigene reporter vector to create hybrid minigene constructs and analyze the resultant mRNA transcripts. To perform the minigene assay, we generated fragments containing exon 12 (where the variant was located) and 150–200 bp flanking intronic regions with XhoI/BamHI restriction sites, which were amplified by PCR from the genomic DNA of the proband. The forward primer contained a XhoI site: 5′accagaattctggagctcgagTGAGTGTCCCCAGGTCTAT and the reverse primer contained a BamHI site: 5′tcaccagatatctgtggatccAGAAGCCGACCCTGAGATTC. The pSPL3 vector was digested by restriction enzymes XhoI/BamHI and then ligated with the purified PCR products. This allowed for construction of the wild-type (WT) and mutant (c.1248+1G>T) minigene vectors using the ClonExpress II One Step Cloning Kit (Vazyme Biotech Co., Ltd.). All constructs were confirmed using bidirectional sequencing.

### Minigene Splicing Assay in HEK293 Cells

HEK293 cells were cultured in 12-well plates with 1 ml of DMEM in each well at 37°C in 5% CO_2_. When the confluence was 80–90%, cells were transfected with 1 μg of purified pSPL3, E12-WT, and c.1248+1G>T plasmids using the Lipofectamine 2000 Transfection Reagent (Thermo Fisher Scientific, Waltham, MA, USA). After 24 h, total RNA was extracted using TRIzol (Thermo Fisher Scientific). The first cDNA strand was reverse transcribed using the HiScript III RT SuperMix for qPCR (Vazyme Biotech Co., Ltd.). The resulting cDNA was used as a template to amplify exon 12 with the SD6 forward primer (5′-TCTGAGTCACCTGGACAACC-3′) and SA2 reverse primer (5′-ATCTCAGTGGTATTTGTGAGC-3′). Reverse transcription PCR (RT-PCR) amplification of aberrant splice transcripts, agarose gel separation, and subsequent direct Sanger sequencing were then performed.

## Results

### Genetic Analysis

We identified a *de novo* classical donor site variant (c.1248+1G>T) in the proband (Figure 1B) which was not detected in his parents (Figure 1A). This variant has not been reported in the genomic databases and was not found in 200 healthy, matched controls. According to the ACMG criteria, the c.1248+1G>T variant was classified as Pathogenic (PVS1+PS2+PM2). Based on the *in-silico* analysis, the c.1248+1G>T variant was predicted to disturb the normal splicing of exon 12.

### Transcript Analysis *in Vivo* and *in Vitro*

**Figure 2:**
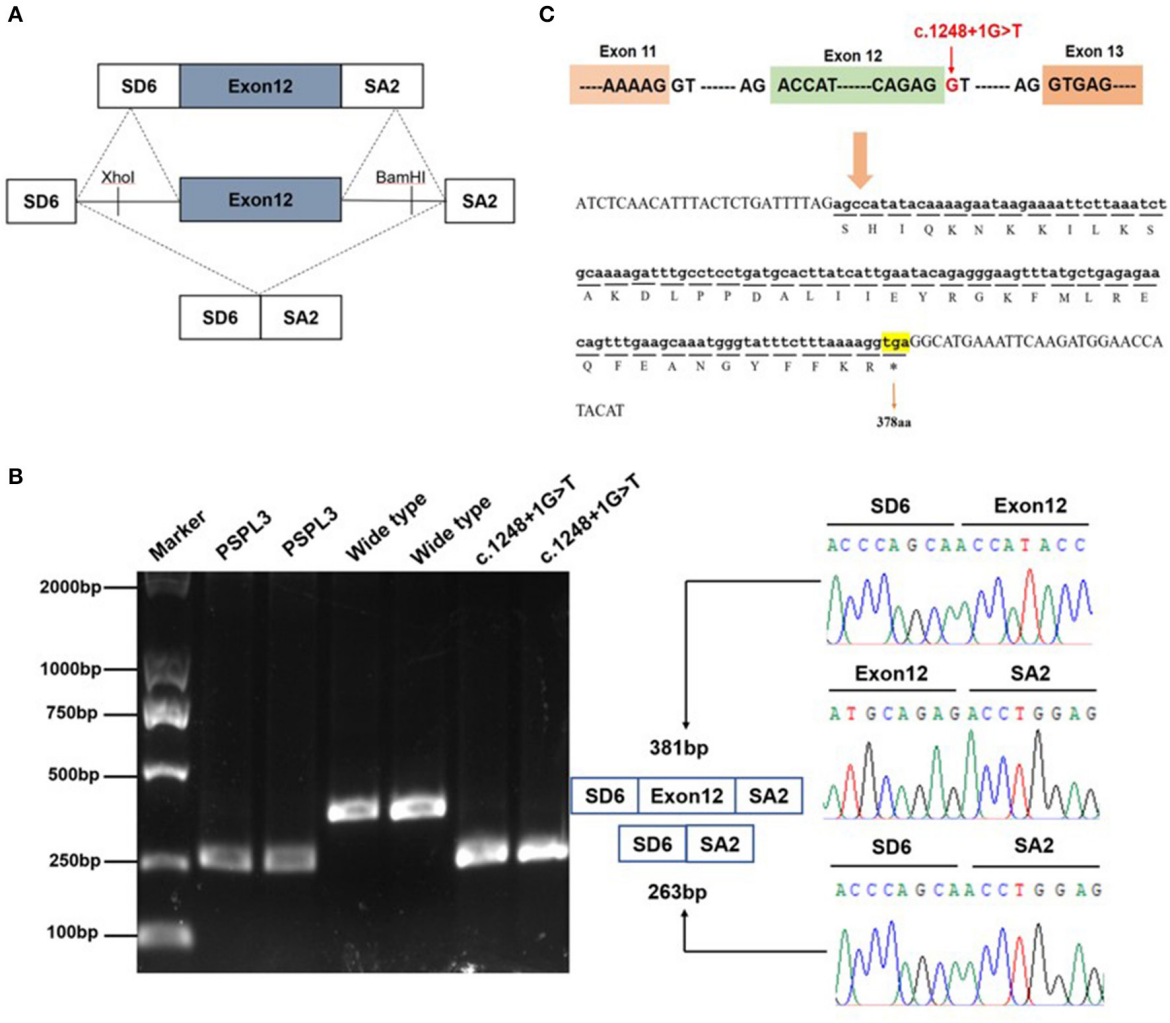
Effect of the *KMT2E* gene c.1248+1G>T variant determined by minigene assays. **(A)** The pSPL3 vector contains 2 exons SD6 and SA2, and a functional intron. All of the indicated fragments were separately cloned into the XhoI and BamHI cloning sites of the pSPL3 vector. SD6 and SA2 primers were designed for RT-PCR amplification of cDNA sequences generated by transfected HEK293 cells. **(B)** Gel electrophoresis of the RT–PCR products of minigene transcripts in HEK293 cells. Lane 1: marker; Lane 2 and 3: pSPL3 (263 bp); Lane 4 and 5: E12-wide type (381 bp); Lane 6 and 7: c.1248+1G>T (263 bp). The two fragments were directly sequenced (right panel). Sequencing analysis of the cDNA showed that the shorter transcript lacked a sequence corresponding to exon 12 of the *KMT2E* gene. **(C)** Exon 12 and adjacent structures in the *KMT2* gene. The arrow shows the location of the c.1248+1G>T splice variant and its effect at the amino acid level. The variant caused by a premature stop codon at amino position 378.

To determine whether the c.1248+1G>T variant affected mRNA splicing *in vivo*, we performed RT-PCR experiments using RNA extracted from peripheral leukocytes. We did not detect any band in the proband, while his father and mother both had a strong *KMT2E* 440 bp product of exon 11 to exon 13 (Supplementary Material). To further clarify the pathogenicity of the c.1248+1G>T variant, we performed *in vitro* RT-PCR splicing validation by constructing E12-WT and mutant (c.1248+1G>T) minigene vectors transfected onto HEK293 cells. The E12-WT construct produced a full-length transcript of the expected size (381 bp) sequence and structure (SD6, Exon 12, and SA2). In contrast, the c.1248+1G>T construct produced a smaller transcript of 263bp containing SD6 and SA2 (Figures 2A,B). Sequence analysis then revealed that the c.1248+1G>T mutant minigene causes aberrant splicing, resulting in the exclusion of exon 12. *In-silico* analysis showed that the mutant transcript created a premature stop codon at 378 amino acids [p.(R378^*^)] could be produced a truncated protein (Figure 2C). Thus, we could not amplify any *KMT2E* mRNA transcript in the proband could be the c.1248+1G>T variant splicing variant cause mRNA degradation.

## Discussion

In this study, we describe the case of a male proband presenting with an onset of FCS, speech delay and ID during childhood, with abnormalities detected on EEG and brain MRI. Informed by this clinical picture, further genetic testing identified a novel *KMT2E* gene splice variant (c.1248+1G>T) as a cause of ODLURO syndrome. We also summarize the clinical information and genetic testing results of 16 patients with ODLURO syndrome in Table 1.

**Table 1:** Clinical manifestation of 17 patients with epilepsy diagnosed with ODLURO syndrome.

**Individual**	**Sex**	**Age**	**Variant**	**Epilepsy**	**Autism**	**Delay**	**Inheritance**	**ID**	**Reported**
**1**	Male	6 y	c.1248+1G>T	Yes	No	Yes	*De novo*	Yes	No
**2**	Female	21 y	c.1239delC (p.Asn414Metfs*4)	Yes	Yes	Yes	Unknown	Moderate	Yes
**3**	Female	3 y,6 m	c.1776_1780delAAAGA(p.Lys593Argfs*17)	Yes	No	Yes	*De novo*	Yes	Yes
**4**	Female	9 y	c.3554C>G(p.Ser1185*)	Yes	No	Yes	*De novo*	Mild	Yes
**5**	Female	36 y	c.4126C>T (p.Pro1376Ser)	Yes	No	Yes	*De novo*	Mild	Yes
**6**	Female	2 y,11 m	c.2720A>T (p.Asp907Val)	Yes	No	Yes	*De novo*	Severe	Yes
**7**	Male	2 y,5 m	c.850T>C (p.Tyr284His)	Yes	NA	Yes	*De novo*	Severe	Yes
**8**	Male	16 y,3 m	c.418G>A (p.Val140Ile)	Yes	Yes	Yes	*De novo*	NA	Yes
**9**	Male	7 y	7:104099959-107002808x1, 2.9 Mb	Yes	No	Yes	*De novo*	Mild	Yes
**10**	Male	22 y	7:103679146-105547471x1, 1.87 Mb	Yes	No	Yes	*De novo*	Moderate	Yes
**11**	Female	18 y	7:104678742-104730547x1, 0.052 Mb	Yes	No	Yes	*De novo*	Moderate	Yes
**12**	Female	1 y	c.5417C>T(p.Pro1806Le)	Yes	No	Yes	*De novo*	Profound	Yes
**13**	Male	4 y	c.186G>A	Yes	Yes	Yes	*De novo*	Mild	Yes
**14**	Male	5 y	chr7:g.104747968G >C(p.Asp1022Hi)	Yes	Yes	Yes	*De novo*	Mild	Yes
**15**	Male	54 y	c.549del(p.Asn183LysfsTer33)	Yes	No	Yes	*De novo*	Mild	Yes
**16**	Female	24 y	c.549del(p.Asn183LysfsTer33)	Yes	No	Yes	Paternal	Mild	Yes
**17**	Male	19 y	c.549del(p.Asn183LysfsTer33)	Yes	No	Yes	Paternal	Severe	Yes

*Individual 2–11 are from reference (1); Individual 12–13 are from reference (13); Individual 14 is from reference (15); Individual 15–17 are from reference (14)*.

The novel *KMT2E* gene splicing mutation (c.1248+1G>T) described in this study resulted in the exclusion of exon 12, and created a premature stop codon at amino acid position R378^*^, producing a truncated protein. Interestingly, no splicing mutations were found in the 16 patients with ODLURO syndrome, which was mainly caused by *KMT2E* missense or deletion mutations. KMT2E is mainly divided into three structures, i.e., a SET/PHD (1–561 aa), central (562–1,122 aa), and C-terminal (1,113–1,858 aa) domain (8, 9). We found that the *KMT2E* c.1248+1G>T mutation is located on the SET/PHD domain. In recent years, it has been reported that the SET domain indirectly affects H3K4, thereby regulating transcription, and the PHD domain is often thought to be related to ID (10, 11). Therefore, we speculate that the location of the c.1248+1G>T mutation in the SET/PHD domain explains why it is dissimilar to other splicing mutations.

Epilepsies associated with *KMT2E* mutations occurred in both male and female patients. However, we found that patients with different mutation types presented with different clinical phenotypes. The seizures of the patient described in this study initially showed a right-sided FCS, which later progressed to a generalized tonic-clonic seizure (GTCS). During the course of the disease, there was only one occurrence of status epilepticus (SE) with fever. After anti-seizure medications (ASMs) were used, the frequency of epileptic seizures was reduced by more than 50%. In contrast to previous reports, four patients with *KMT2E* missense variants presented with GTCS at the beginning of the disease, and multiple SE occurred over the course of the disease. In these cases, treatment with more than three ASMs was ineffective. In cases of intractable epilepsy (IE), the ketogenic diet (KD) is also ineffective. Therefore, we found that children with missense variants present with more overt clinical phenotypes. Patients with missense variants might also have a higher likelihood of developing IE (1, 12). Previous studies have shown that missense variants may gain more functions or a dominant negative effect, leading to more severe clinical features (13, 14). This difference is also reflected in the bilateral central and midline area small spines and background slow waves evident for the proband on EEG. This stands in contrast to high arrhythmias and bursts in patients with other missense variants. Therefore, patients with *KMT2E* gene missense variants will not only have more serious clinical manifestations, but EEG findings will also be consistent with more serious burst suppression waves.

The physical examination of the patient reported in this study revealed a smaller head circumference. About 50% of the cases reported by O 'Donnell-Luria et al. had macrocephaly, microcephaly only occurs in two patients with microdeletion (1). We also found very low IQ (Intelligence Quotient) in these two patients and hypothesized that this very low cognitive impairment is related to cerebral atrophy. The brain MRI of proband suggested the delay of myelination, consistent with cerebral atrophy evident in *KMT2E* missense carriers. The proband's brain MRI was normal at the onset of the disease, but delayed myelination appeared 1 year after the onset. We speculated that this change was due to the extensive expression of KMT2E gene in the brain.The specific *KMT2E* NKP44L (Natural Killer Cell No. 44 Receptor Ligand) subtype is known to regulate NKp44-NKp44L-mediated human natural killer cells and other innate lymphocytes (15, 16). According to the “Human Protein Atlas,” this protein is expressed in the prefrontal cortex, spinal cord, and cerebellum as an activator of the NKp44-NKp44L-mediated immune response (17). Therefore, the *KMT2E* mutation may cause increased NKp44L expression on the surface of neuronal cells, inducing a NKp44-NKp44L-mediated immune response, which in turn leads to abnormal brain activity associated with illness. We thus speculate that abnormal brain development in patients with ODLURO syndrome is driven by *KMT2E* mutations.

Since there are no specific drug treatments for epilepsy in patients with ODLURO syndrome, the focus is confined to symptomatic management. We used traditional ASMs to control patients' seizures. However, this is ineffective in some patients, and the development of alternative treatments constitutes an important focus of ongoing research. In recent years, some researchers have proposed that ion channel modulators are more suitable for the treatment of epilepsy in patients with ODLURO syndrome. The *KMT2E* gene is translated to a trithorax (TrxG) protein of 1,858 amino-acid residues. The product of *Trxg* gene expression can stimulate the tyrosine kinase (BAP) protein subunit of the recombinant human ubiquitin (SUMO) coupling enzyme to promote protein assembly, recruitment, or remodeling. Therefore, mutations in the *KMT2E* gene may affect the function of certain ion channel genes, leading to loss of function, imbalances in neuronal cell membrane potential, and induction of abnormal cerebral wave emission, leading to epilepsy. The use of ion channel blockers might therefore be an effective treatment option for managing epileptic seizures in patients with ODLURO syndrome.

## Conclusion

In summary, this study describes the identification of a novel *KMT2E* splice variant (c.1248+1G>T) in a male patient diagnosed with ODLURO syndrome. This variant can impair splicing of the donor site in causing mRNA exclusion and the production of a truncated protein. In conclusion, the genetic diagnosis of *KMT2E* can be used to guide the prenatal diagnosis, clinical management, and prognostication of ODLURO syndrome.

## Data Availability Statement

The original contributions presented in the study are publicly available. This data can be found in the LOVD database: https://databases.lovd.nl/shared/individuals?create, ID00388513.

## Ethics Statement

Written informed consent was obtained from the individual(s), and minor(s)' legal guardian/next of kin, for the publication of any potentially identifiable images or data included in this article.

## Author Contributions

JC and CW conceived, designed this study, reviewed, and edited the manuscript. ZC and CW wrote the manuscript and performed the experiments. ZC and JC collected the clinical samples, clinical data, and wrote the clinical part of the manuscript. CW, GZ, LD, and HG performed NS analysis. All authors contributed to the article and approved the submitted version.

## Funding

This work was supported by medicine science and technology development foundation, Nanjing Municipal Health bureau (ZKX19038).

## Conflict of Interest

The authors declare that the research was conducted in the absence of any commercial or financial relationships that could be construed as a potential conflict of interest.

## Publisher's Note

All claims expressed in this article are solely those of the authors and do not necessarily represent those of their affiliated organizations, or those of the publisher, the editors and the reviewers. Any product that may be evaluated in this article, or claim that may be made by its manufacturer, is not guaranteed or endorsed by the publisher.
