# Supplementary material for: Case Report: A Novel KMT2E Splice Site Variant as a Cause of O'Donnell-Luria-Rodan Syndrome in a Male Patient
Source: Front Pediatr. 2022 Feb 22;10:822096. doi: 10.3389/fped.2022.822096 (PMC8901719; doi:10.3389/fped.2022.822096)
Supplement: Supplementary file 1 [file Data_Sheet_1.docx]

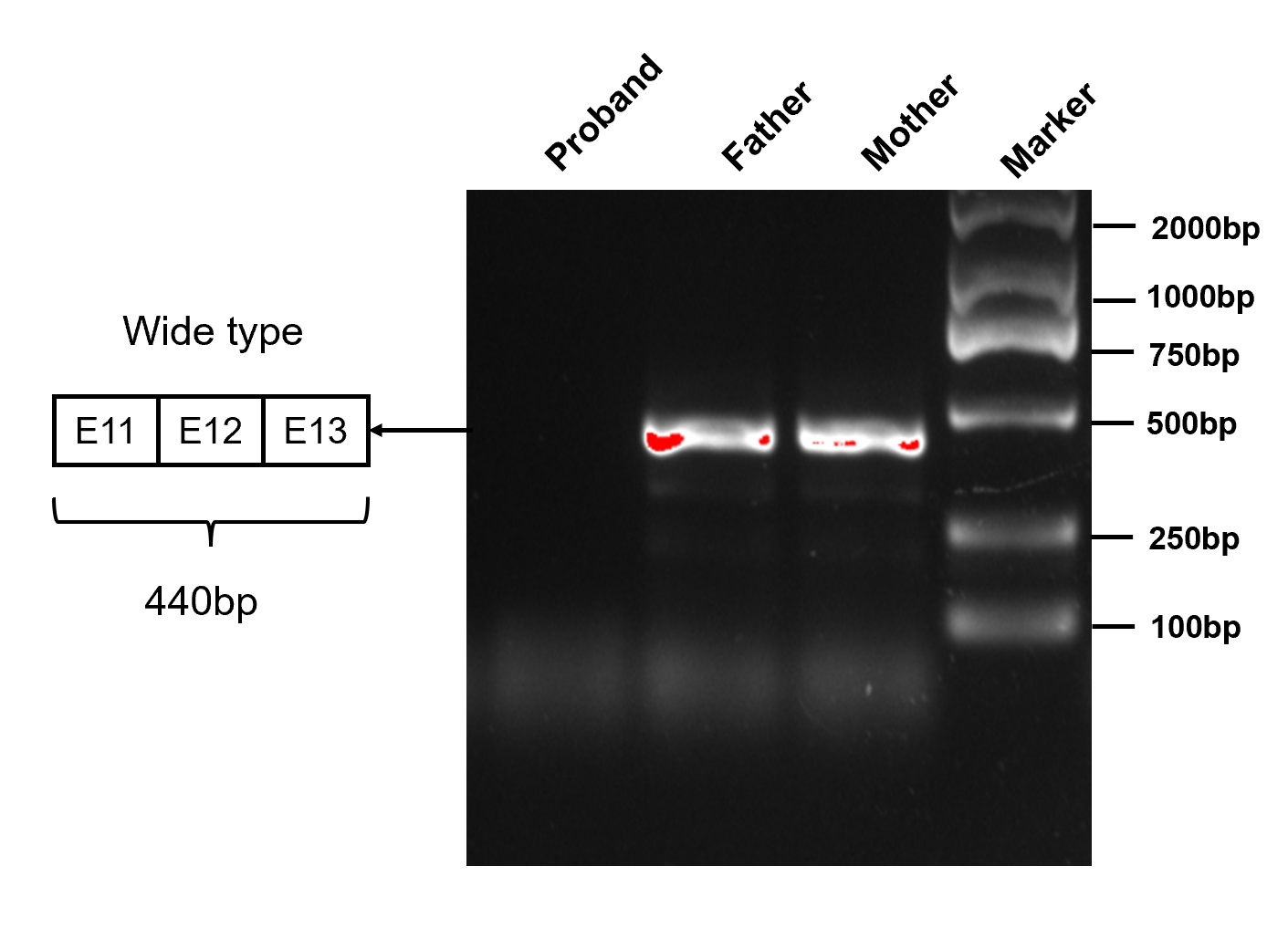


**Figure legends**: Gel electrophoresis of RT-PCR fragments in vivo showed that the proband did not detect any KMT2E mRNA transcript while his healthy parents detected specific transcript (440bp) containing exon11-exon13 .
